# Use of Mercury in Dental Silver Amalgam: An Occupational and Environmental Assessment

**DOI:** 10.1155/2016/6126385

**Published:** 2016-06-30

**Authors:** Nadia Jamil, Mujtaba Baqar, Samar Ilyas, Abdul Qadir, Muhammad Arslan, Muhammad Salman, Naveed Ahsan, Hina Zahid

**Affiliations:** ^1^College of Earth and Environmental Sciences, University of the Punjab, Lahore 54590, Pakistan; ^2^College of Petroleum and Geosciences, King Fahd University of Petroleum & Minerals, Dhahran 31261, Saudi Arabia; ^3^Institute of Chemistry, University of the Punjab, Lahore 54590, Pakistan; ^4^Institute of Geology, University of the Punjab, Lahore 54590, Pakistan

## Abstract

The objective of this study was to assess the occupational exposure to mercury in dentistry and associated environmental emission in wastewater of Lahore, Pakistan. A total of ninety-eight blood samples were collected comprising 37 dentists, 31 dental assistants, and 30 controls. Results demonstrate that the dentistry personnel contained significantly higher mean concentration of mercury in their blood samples (dentists: 29.835 *µ*g/L and dental assistants: 22.798 *µ*g/L) compared to that of the controls (3.2769 *µ*g/L). The mean concentration of mercury was found maximum in the blood samples of older age group (62.8 *µ*g/L) in dentists and (44.3 *µ*g/L) in dental assistants. The comparison of mercury concentration among dentists, dental assistants, and controls (pairing based on their ages) revealed that the concentration increased with the age and experience among the dentists and dental assistants. Moreover, the mercury concentration in all the studied dental wastewater samples, collected from twenty-two dental clinics, was found to be exceeding the recommended discharge limit of 0.01 mg/L. Therefore, we recommend that immediate steps must be taken to ensure appropriate preventive measures to avoid mercury vapors in order to prevent potential health hazards to dentistry personnel. Strong regulatory and administrative measures are needed to deal with mercury pollution on emergency basis.

## 1. Introduction

Mercury has been used in dental silver amalgam for the last two centuries [1], as a dental restorative filling material. This material contains mercury in about 50% of its total mass and the remaining constituents are silver, tin, copper, zinc, and other trace metals [2]. Though the dental amalgam is widely used, however, its injudicious handling consequently leads to human health risk, particularly associated with occupational exposure and environmental damage from mercury emission [3]. Some alternative filling materials are also available in dentistry but low cost, durability, and easiness in handling and placement have maintained the popularity of the mercury based dental amalgam in most parts of the world, where it is used as filling material in posterior teeth [4, 5]. The development of Minamata and Convention on Mercury, an international treaty adopted by 139 countries, came forward as a major instrument to call a voluntary phase-down of mercury use in dental amalgam [6, 7].

The dentist and their assistants have been occupationally exposed to different forms of mercury across the world [8, 9]. More specifically, elemental mercury vapors (HgO) are considered as a major form due to manipulation of dental amalgam in their several routine occupational tasks, including preparation, restoration, and removal of dental amalgam [10]. Approximately, 80% of the inhaled mercury vapors is captivated in the blood stream, circulates throughout the body, and can pass through both the placental and the blood-brain barriers [4, 11]. Furthermore, dental personnel are also exposed to inorganic mercury (mercuric salts, mercurous compounds) and organomercurials from contaminated diet intake and mercury stemming from their own dental amalgam fillings [11].

In addition to this, studies have also reported that the use of mercury in dentistry is consequently associated with 10–70% of the total daily mercury load in the wastewater collection system [12]. This controversial release of mercury being associated with dentistry has become a matter for concern especially during the last three decades. Although dentists in developed countries have become aware of their environmental responsibilities towards mitigation of adverse impacts associated with amalgam handling and have adopted appropriate measures to confront the matter [13], however, the dentists in less developed countries, particularly in South Asia, are deliberately ignoring the issue. The dental amalgam waste in Pakistan and India is disposed of into wastewater streams diluted or undiluted [14–17]. According to Mumtaz et al. [18], about 92% of dentists in Pakistan used amalgam but also perceived it as a health risk. However, 56% of the subjects disagreed that amalgam should be replaced with nonmercury fillings. Therefore, the aim of this study is to assess the mercury accumulation in the blood stream of the dental personnel and its discharge into environment from the private dental practitioner's clinics from Lahore, Pakistan, a signatory country to Minamata Convention of Mercury (2013), which restricts the use and emission of hazardous mercury.

## 2. Materials and Methods

### 2.1. Study Population and Working Conditions

A total of 98 individuals comprising 37 dentists, 31 dental clinic assistants, and thirty control group individuals were sampled randomly, covering the diverse environment of Lahore City. Sampling took place in March and April 2015. Each individual was questioned and interviewed to get the information about their daily routine on the following variables, that is, age, gender, working hours, years of experience, nutrition habits (especially frequency of fish consumption), smoking, and amalgam filling per week (Table 1). A basic medical examination of every individual was performed, investigating the dental status especially numbers of amalgam fillings by a local dentist. The control group individuals were university academic professionals and students.

### 2.2. Samples Collection and Preparation

#### 2.2.1. Blood Samples

Five milliliters of venous blood was collected in metal-free vacutainers. The blood samples were centrifuged at 1500 ×g for 20 minutes at 5°C. The packed erythrocytes and plasma were separated by means of a serum separator while plasma samples were subjected to mercury determination for further analysis [19]. The samples were wet-digested with perchloric acids and nitric acids (1 : 5) at 25–35°C followed by filtration by Whatman Ashless Filter Paper 90 mm Ø and, finally, added to bidistilled water to make a total volume of 10 mL. Thereafter, wet-digested samples were subjected to mercury content determination using inductively coupled plasma/optical emission spectrometer (ICP-OES), Perkin-Elmer Optima 2000 DV, in triplicate while maintaining the variation between three runs as low (CV < 10%). The accuracy of the method was validated by adding predetermined amounts of Hg^+2^ in HNO_3_ to other blood plasma samples to roughly double the original mercury concentration. All the measurements were assessed in *µ*g/L and expressed in terms of total blood mercury, considering the plasma and erythrocyte ratio, 2 : 3 [20].

Finally, the whole population was analyzed against three levels of mercury as suggested by Mayo Medical Laboratories according to the exposure and effects. These levels are normal as 0–9 *µ*g/L (<10 *µ*g/L), individuals with mild exposure such as dentists as 10–15 *µ*g/L, individuals with high exposure such as patients as 15–50 *µ*g/L, and individuals with significantly higher exposure when the whole blood mercury level is >50 *µ*g/mL (test ID: HG-8618).

#### 2.2.2. Wastewater Samples

The wastewater samples were obtained from twenty-two dental clinics at the end of the working day having no mercury separation technique. The sampling was performed at two points, that is, the discharge point of dental wastewater into municipal wastewater collection system (grab samples) and the side-holding tank attached to dental chairs (mostly composite samples). Three replicate samples were collected from each sampling point on three consecutive working days. All the wastewater samples were collected and preserved in accordance with the standard methods of the American Public Health Association [21]. The wastewater samples were first digested using potassium permanganate and potassium peroxodisulfate solution. In the digested sample, hydroxylammonium chloride solution was added, followed by addition of tin(II) chloride, the reducing agent [22]. The mercury concentration in samples was determined by using ICP-OES (Perkin-Elmer Optima 2000 DV). Standard stock solution of mercury with concentration of 1000 ppm (J/8047/08), initially prepared by the Fisher Scientific, was used in this study.

### 2.3. Quality Control

The accuracy of mercury analysis was assessed using advanced mercury analyzer by running samples in triplicate. Recovery varied between 92.3 and 101.4%. A good agreement was found between the obtained mean and the certified value. Furthermore, 15% of the randomly selected samples were analyzed thrice in order to evaluate the reproducibility.

### 2.4. Statistical Analysis

The STATISTICA 7.0 software (Stat Soft, Inc., 2004) was employed to perform the statistical analysis. The descriptive statistical parameters such as arithmetic mean, standard deviation, and the respective confidence limits were calculated for the blood mercury content and one-way ANOVA (*p* < 0.05) was performed. Among questionnaire variables, age, sex, working hours, years of experience, and number of amalgam filling were considered as independent variables, while mercury concentration in blood samples was considered as a dependent variable. The data was tested for the assumption of normality using the Kolmogorov-Smirnov test. In the end, the Correspondence Analysis (CA) was performed to describe the relationships of age groups with different levels of mercury as risk/exposure factor among dentists and dental assistants.

## 3. Results and Discussion

### 3.1. Occupational Exposure Assessment

The descriptive statistics of mean mercury concentration in the blood samples of dentists, dental assistants, and controls is summarized in Table 2. One-way ANOVA was applied on the data; related individual's age, working classes, working hours, experience, dental filling per week, person's own amalgam filling, and number of own fillings were found statistically significant (Table 3), whereas the smoking and feeding habits were nonsignificant (*p* < 0.05).

**Figure 1:**
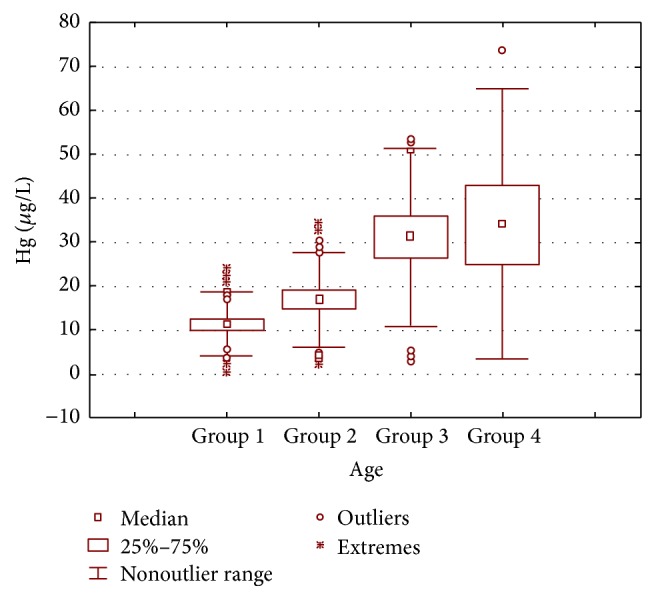
Box and whisker plot of mean mercury concentration (*µ*g/L) in the blood samples of dentists, dental assistants, and controls according to their age groups.

There was a gradual increase in accumulation of mercury concentration with age among dental personnel. The highest mean mercury concentration (62.833 *µ*g/L) was recorded in group 4 (51–60 years) (Figure 1). The investigation about the mercury level within groups revealed that the magnitude of mercury among dentists and dental assistants was found to be in the order* group 4 > group 3 > group 2 > group 1*. Group 4 had the highest level of mercury concentration and, hence, possesses significant risk potential compared to group 3. Likewise, group 3 possesses greater risks compared to group 2 and so on. This statistical significance of age parameter on mercury levels in individuals has been in consistence with previously reported findings [9, 23, 24] and in contrast to [25–27].

**Figure 2:**
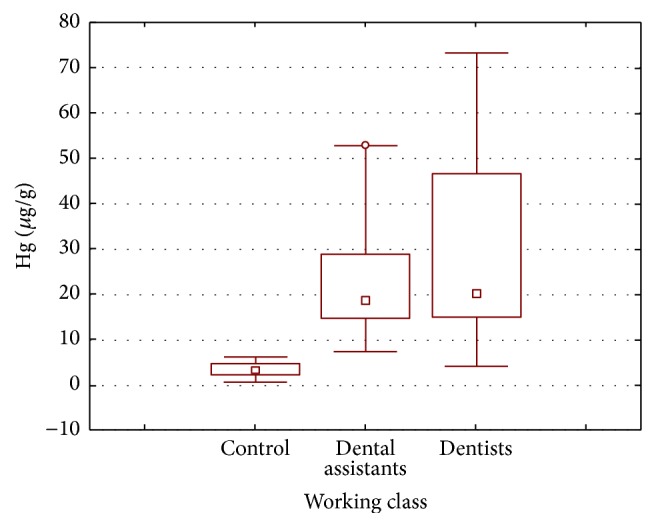
Box and whisker plot of mean mercury concentration (*µ*g/L) in the blood samples of dentists, dental assistants, and controls according to their working class.

Among the three working classes, that is, dentists, dental assistants, and controls (Figure 2), the maximum mean concentration was recorded in dentists (29.835 *µ*g/L), followed by dental assistants (22.798 *µ*g/L) and controls (3.276 *µ*g/L). These high levels of mercury in dental personnel indicate the chronic accumulation of mercury in the blood of dentists and dental assistants due to their occupational exposure of elemental mercury vapors. A study from Pakistan revealed that 100% of the studied private dental clinics have significantly higher levels of mercury vapors in indoor air than ATSDR limit [17]. The inhaled mercury vapors move into blood stream from lungs and circulate in the human body, affecting different organs and systems [28, 29]. Contrary to the findings of Langworth et al. [23], the mercury concentrations in dentist blood samples are found to be higher than those of dental assistants in this study. Though the dentists and dental assistants both are occupationally exposed to mercury vapors [30], the highest levels of mercury in dentists might be associated with the fact that the dentists are directly involved in the amalgam filling process at workplace and are relatively more exposed to the mercury vapors than dental assistants, who generally spend less time in mercury exposure. Of the inhaled mercury vapors, about 80% of the mercury vapors are retained in the circulating red blood cells [4]. So the mercury levels are relatively high in blood samples of dentists.

**Figure 3:**
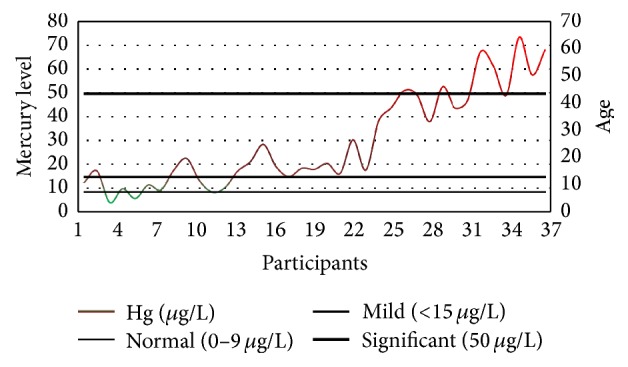
The level of blood mercury concentrations among dentists along with their ages.

According to the Mayo-derived standards, as explained previously, only five dentists (14%) were found to have mercury concentration lower than the devised limit, that is, 10 *µ*g/L; however, the concentration was significantly higher in the remaining population, that is, 32 dentists (86%). Among these dentists, five individuals (16%) were found to have mercury concentration ranging within 10–15 *µ*g/L (mild exposure); 20 individuals (62%) had mercury concentration within 15–50 *µ*g/L (high exposure); and 7 individuals (22%) were found to have mercury concentration significantly higher than 50 *µ*g/mL (significantly high exposure). The complete illustration is provided in Figure 3 using the idea of gradient color process control charts where the mercury levels are presented in terms of upper control limits, that is, normal, mild, and significant.

**Figure 4:**
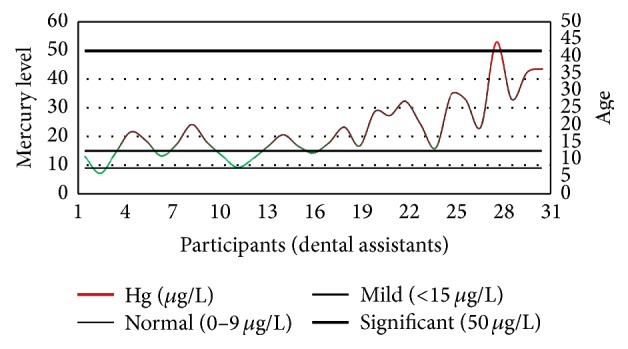
The level of blood mercury concentrations among dental assistants along with their ages.

**Figure 5:**
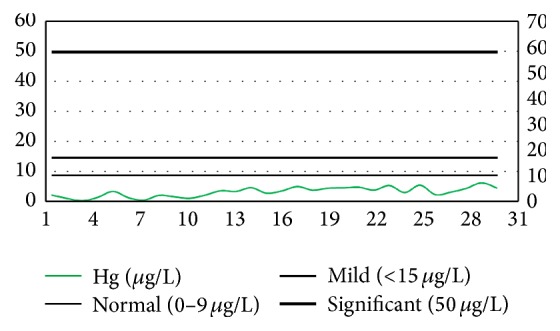
The level of blood mercury concentrations among control group along with their ages.

Among dental assistants, only 2 individuals (7%) had mercury concentration within the range, whereas 6 individuals (19%) ranged within 10–15 *µ*g/L (mild exposure); 22 individuals (71%) ranged within 15–50 *µ*g/L (high exposure); and 1 individual (3%) was found to have mercury concentration higher than the 50 *µ*g/L (significantly high exposure) (Figure 4). However, there was no well-defined pattern of mercury concentration observed in control group population (Figure 5).

**Figure 6:**
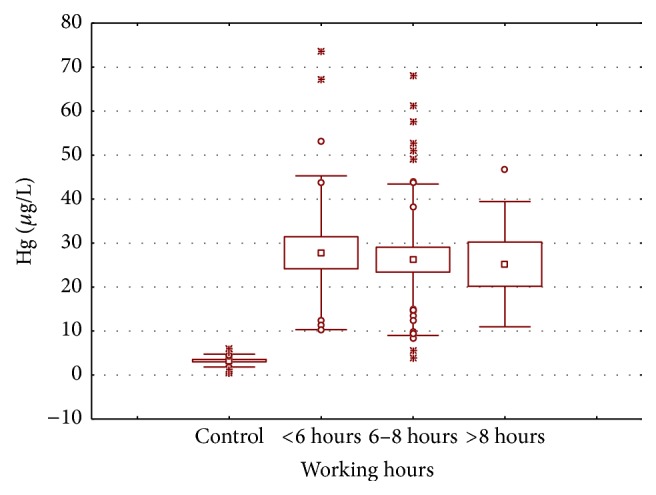
Box and whisker plot of mean mercury concentration (*µ*g/L) in the blood samples of dentists and dental assistants according to their daily working hours.

**Figure 7:**
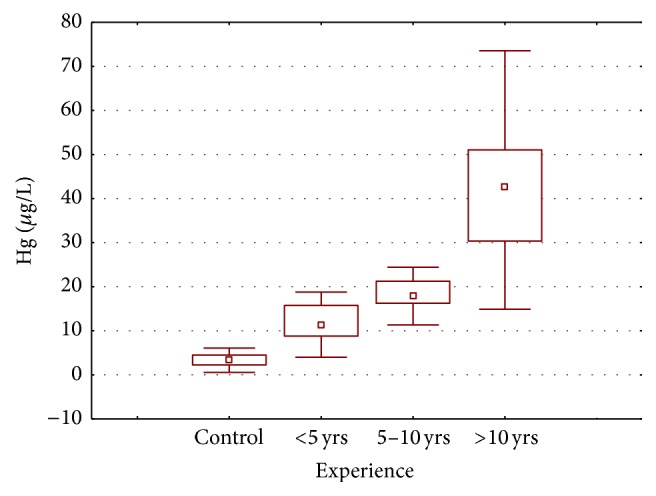
Box and whisker plot of mean mercury concentration (*µ*g/L) in the blood samples of dentists and dental assistants according to their work experience.

Higher blood mercury content was also significantly associated with dental personnel's daily working hours (Figure 6) which is inconsistent with those reported from Kasraei et al. [24] and Ritchie et al. [26]. In addition to the age and daily working hours' effect on mercury accumulation, similar pattern of mercury distribution was observed for work experience (Figure 7), where the mean mercury content was found to be greater in dental personnel with relatively more work experience. The highest mean mercury level (41.556 *µ*g/L) was found in personnel with more than 10 years' work experience followed by those with 5–10 years' work experience (18.154 *µ*g/L), and the lowest was found in those with less than five years' work experience (11.749 *µ*g/L). Karahalil et al. [27] and Baelum and Pockel [31] have also reported that the mercury concentration increases in dentist's body with increase in work experience.

**Figure 8:**
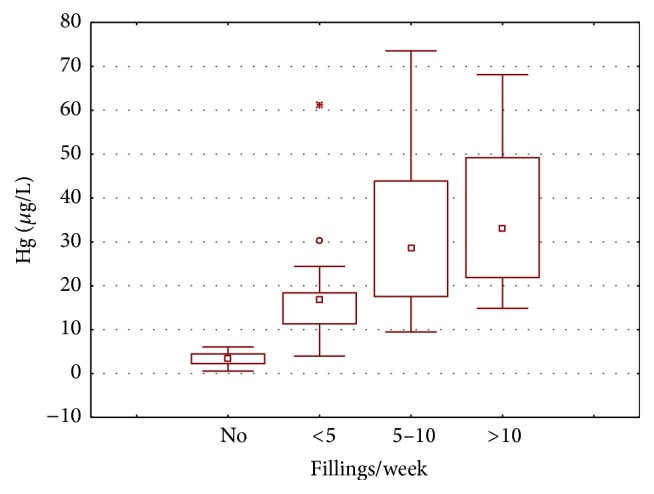
Box and whisker plot of mean mercury concentration (*µ*g/L) in the blood samples of dentists and dental assistants according to amalgam filling performed per week.

The number of amalgam fillings per week performed by dental personnel has shown an increasing trend for mercury concentration in blood samples (Figure 8). The highest mean mercury concentration (36.510 *µ*g/L) was recorded in personnel with greater than 10 fillings performed per week, followed by 5–10 fillings per week (32.156 *µ*g/L) and less than 5 fillings per week (16.781 *µ*g/L). Ritchie et al. [26] found significant correlation between number of amalgam fillings per week and urinary mercury concentration among dentists. The rise in mercury concentration with increase in number of amalgam fillings is because more mercury vapors are produced with the amount of amalgam filling prepared. A significant correlation was studied between number of dental amalgam fillings per week and mercury vapors concentration at the dental workplace that also results into blood mercury accumulation [3]. This chronic accumulation/exposure of dentists and dental assistants to mercury vapors can cause neurological impairments, hormonal imbalances, and reproductive disorders [11, 32, 33].

**Figure 9:**
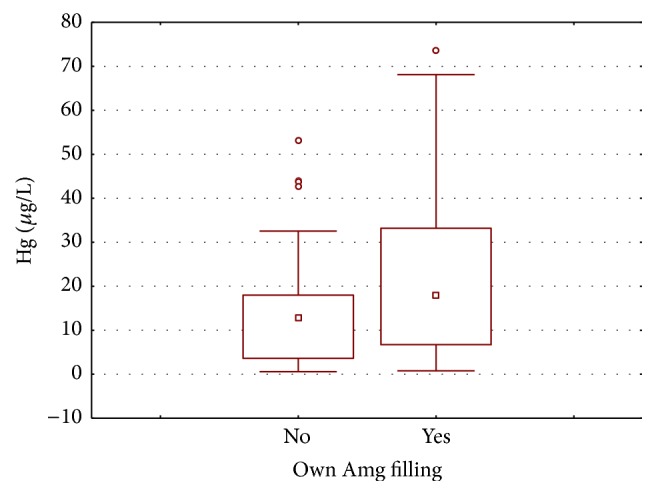
Box and whisker plot of mean mercury concentration (*µ*g/L) in the blood samples of dentists, dental assistants, and controls according to their own amalgam filling.

**Figure 10:**
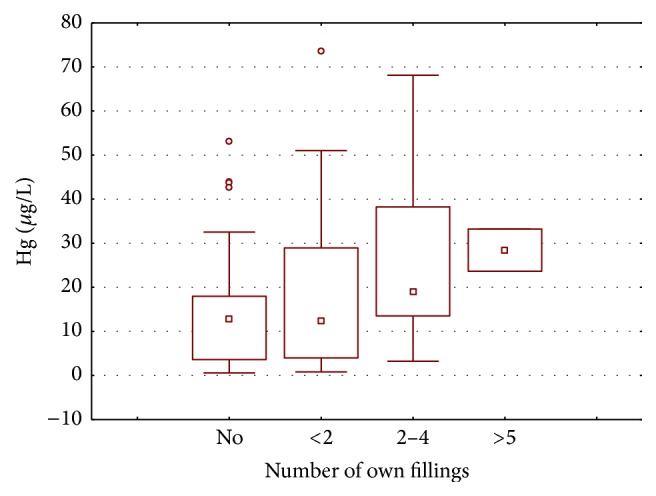
Box and whisker plot of mean mercury concentration (*µ*g/L) in the blood samples of dentists, dental assistants, and controls according to their own amalgam filling.

The subjects with own amalgam filling are found to have mean mercury concentration of 22.902 *µ*g/L, which is relatively higher than the subjects having no amalgam fillings (Figure 9). The results further elucidate that the mean mercury concentrations increase with the number of own amalgam fillings (Figure 10), the highest mean concentration being in individuals with greater than five amalgam fillings (28.422 *µ*g/L), followed by those with two to five amalgam fillings (26.440 *µ*g/L), and the lowest being among those with less than two amalgam fillings (19.463 *µ*g/L).

Overwhelmingly, the mercury concentrations in all the groups of dentists and dental assistants were significantly higher than in the control group. A similar trend was observed by different studies, whereby the mercury concentration in dentists was higher than in those individuals not occupationally exposed [9, 19, 26]. One reason behind this fact could be that the inorganic mercury entrapped in the red blood cells is ultimately removed from the body with disintegration of red blood cells in the bile salts [34]. Therefore, the nonoccupationally exposed groups (controls) may not be able to sustain higher concentrations of mercury.

### 3.2. Wastewater Assessment

The mercury concentrations released into the environment through wastewater discharges from dental clinics are depicted in Table 4. The highest and lowest mean mercury levels (±SD) assessed were 261 mg/L (±83.431) and 86.667 mg/L (±56.224) in samples obtained from effluent discharge point into wastewater collection system. In the samples collected from the side-holding tank of dental chairs, the highest and lowest mean mercury levels (+SD) were 343.333 mg/L (±45.716) and 112 mg/L (±23.245), respectively. The mercury and mean mercury concentrations in discharge point samples were relatively lower than side-holding tank samples (Table 4). The relatively low concentrations were probably due to the dilution with effluent of nonamalgam activities in dental clinics as dilution would decrease heavy metal concentrations [35–37].

Mercury concentrations in wastewater discharge point samples are of a similar magnitude as those reported by Welland [38] and were exceeding the local discharge limits of 0.01 mg/L in all the study dental wastewater samples [39]. It is reported that the dental wastewater can generate up to 4.5 g Hg/day/chair [40, 41] and an estimated 100–200 g of mercury per year per dental office [42]. No particular legislation, predominantly in the developing countries, including the study country, is associated with discharge of mercury from small dental clinics. Studies have reported that the nonregulated clinic wastewater may contribute up to 70% of the total mercury daily load to the municipal wastewater facilities [12].

This high release of mercury into the environment in the present study is mainly associated with residual noncontact amalgam and waste amalgam from filling removal, with no mercury recycling and/or separation activities involved. The residual noncontact amalgam can be easily recycled with generation potential of 211 mg of mercury/day/chair [43], which would otherwise have been disposed of in the municipal wastewater collection system. However, several techniques are introduced in the market to separate the mercury content from the dental wastewater. Drummond et al. [43] have reported that the filtration, gravity settling, and ion exchange techniques can remove 93.4–98.8%, 99.3%, and 79.0% of the total wastewater mercury content, respectively. The inorganic mercury from the dental wastewater can undergo methylation by bacteria and fungi to produce methylmercury up to 26.77 *µ*g/L in dental wastewater [44]. The methylmercury is of great concern for aquatic ecosystem and public health as it is a potential neurotoxin that bioaccumulates in the muscular tissues of fish and undergoes biomagnification as it moves to human through the food chain [45]. The high level of mercury release into environment through dental wastewater would affect the biosphere, particularly the riverine aquatic ecosystem as the river is the ultimate wastewater disposal point of the study region [46]. Thus, the mercury content being released from dental clinics may develop the dangerous levels of mercury in local fish.

## 4. Conclusions

The mercury concentrations in dental personnel are found to be significantly elevated than in controls, with the highest mean concentrations recorded in older and experienced dentists. The total mercury concentrations in all the dental wastewater samples were also exceeding limits in all samples. Thus, based on the present study, instantaneous steps shall be taken to safeguard the health of the dentists and dental assistants through appropriate preventive measures for mercury vapors by utilization of alternative filling material. As far as wastewater discharges are concerned, it is recommended to ensure the implementation of particular legislation and to deal with mercury in wastewater in Pakistan.

## Figures and Tables

**Table 1:** Sociodemographic characteristics of the dentists, dental assistants, and controls.

Characteristics	Dentists (*n* = 37)		Dental assistants (*n* = 31)		Controls (*n* = 30)	
	*n*	%	*n*	%	*n*	%
Age (years)						
21–30 (group 1)	12	32	19	55	11	36
31–40 (group 2)	11	30	13	29	8	27
41–50 (group 3)	8	22	5	16	6	20
51–60 (group 4)	6	16	0	0	5	17
Sex						
Male	33	89	31	100	27	90
Female	4	11	0	0	3	10
Smoking						
Yes	8	22	18	58	11	37
No	29	78	13	42	19	63
Working hours						
Less than 6	14	38	9	29	—	—
6–8	21	57	16	52	—	—
More than 8	2	5	6	19	—	—
Years of experience						
Less than 5	7	19	9	29	—	—
5–10	11	30	12	39	—	—
More than 10	19	51	10	32	—	—
Frequent fish consumption^*∗*^						
Yes	12	32	3	10	10	33
No	25	68	28	90	20	67
Amalgam filling per week						
<5	16	43	11	36	—	—
5–10	17	46	15	48	—	—
>10	4	11	5	16	—	—
Own amalgam filling						
Yes	27	73	19	61	14	47
No	10	27	12	39	16	53
Number of fillings^*∗∗*^						
<2	16	59	6	32	9	64
2–5	11	41	11	58	5	36
>5	0	0	2	10	0	0

^*∗*^Fish consumption at least once a week.

^*∗∗*^Applicable to subjects with their own amalgam filling.

**Table 2:** Mean mercury concentration (*µ*g/L) in the blood samples of dentists, dental assistants, and controls.

	Sample size (*n*)	Mean Hg (*µ*g/L)	Standard deviation
*Dentists* (*n* = 37)			
Group 1	12	11.8043	5.2648
Group 2	11	20.1561	4.91
Group 3	8	45.4438	5.5091
Group 4	6	62.8332	8.7466
*Dental assistants* (*n* = 31)			
Group 1	19	16.4516	4.4547
Group 2	8	27.6555	6.0964
Group 3	4	43.2331	8.1866
Group 4	0	0	0
*Controls* (*n* = 30)			
Group 1	11	1.7691	0.8087
Group 2	8	3.9253	0.6833
Group 3	6	4.4708	0.8654
Group 4	5	4.1234	1.3791

**Table 3:** One-way ANOVA of daily activities and dental health of dentists, dental assistants, and controls population.

	Sum of squares	Degree of freedom	Means of square	*F*	*p*	Significance
Working class	12185.44	2	6092.72	32.049	0.000	*∗*
Daily working hours	11404.47	3	3801.49	18.966	0.000	*∗*
Amalgam fillings/week	15824.88	3	5274.96	34.385	0.000	*∗*
Number of own fillings	2577.81	3	859.27	2.9193	0.038	*∗*
Smoking	139.93	1	139.93	0.446	0.505	NS
Work experience	23006.71	3	7668.9	99.591	0.000	*∗*
Feeding habits	720.77	1	720.77	2.343	0.129	NS

^*∗*^Significant at *p* > 0.05; NS: nonsignificant values.

**Table 4:** Mean mercury concentration (mg/L) in wastewater of dental clinics, from Lahore.

Collection site	Number of chairs	Mean Hg^*∗*^	Mean Hg^*∗∗*^
Clinic A	2	183.0 (±20.7)	237.0 (±33.2)
Clinic B	3	221.7 (±71.7)	264.0 (±22.3)
Clinic C	2	248.3 (±32.2)	279.4 (±76.8)
Clinic D	1	257.0 (±36.1)	299.7 (±32.2)
Clinic E	3	261.0 (±83.4)	313.7 (±19.7)
Clinic F	2	107.0 (±47.9)	161.5 (±36.1)
Clinic G	2	162.7 (±51.8)	259.0 (±65.5)
Clinic H	2	137.3 (±35.6)	211.3 (±15.7)
Clinic I	1	115.7 (±16.8)	186.3 (±29.5)
Clinic J	2	162.0 (±20.7)	237.0 (±25.7)
Clinic K	2	86.7 (±56.3)	112.0 (±23.2)
Clinic L	2	179.0 (±62.2)	251.7 (±42.6)
Clinic M	2	156.3 (±88.3)	278.0 (±9.71)
Clinic N	1	164.3 (±49.7)	197.3 (±12.0)
Clinic O	2	212.7 (±7.02)	343.3 (±45.7)
Clinic P	2	169.0 (±13.0)	242.7 (±29.5)
Clinic Q	2	247.0 (±15.5)	295.0 (±11.1)
Clinic R	3	126.0 (±67.6)	263.7 (±19.8)
Clinic S	2	170.7 (±44.6)	253.3 (±44.5)
Clinic T	2	101.7 (±82.1)	218.3 (±17.9)
Clinic U	2	251.7 (±56.2)	318.0 (±25.3)
Clinic V	1	91.3 (±42.8)	143.0 (±23.2)

*Mean*	*1.95*	*173.3*	*243.9*

*SD*	*0.57*	*56.54*	*58.23*

Each value is the mean of three replicates; the standard deviation of three replicates is presented in parentheses.

^*∗*^Samples collected from discharge point into wastewater collection system.

^*∗∗*^Samples collected from the side-holding tank of dental chairs.
